# Development of a Whole-Virus ELISA for Serological Evaluation of Domestic Livestock as Possible Hosts of Human Coronavirus NL63

**DOI:** 10.3390/v11010043

**Published:** 2019-01-09

**Authors:** Philip El-Duah, Benjamin Meyer, Augustina Sylverken, Michael Owusu, Lina Theresa Gottula, Richmond Yeboah, Jones Lamptey, Yaw Oppong Frimpong, Vitus Burimuah, Raphael Folitse, Olivia Agbenyega, Samuel Oppong, Yaw Adu-Sarkodie, Christian Drosten

**Affiliations:** 1 Department of Clinical Microbiology, Kwame Nkrumah University of Science and Technology, PMB, UPO, Kumasi 00233, Ghana; elduahphilip9@gmail.com (P.E.-D.); Yeboahrichmond82@yahoo.com (R.Y.); jlamptey80@gmail.com (J.L.); yasax@hotmail.co.uk (Y.A.-S.); 2 Kumasi Centre for Collaborative Research in Tropical Medicine, PMB, UPO, KNUST, Kumasi 00233, Ghana; annan@kccr.de (A.S.); owusumichael-gh@hotmail.com (M.O.); oppongfrimpong1@gmail.com (Y.O.F.); vitus7uk@yahoo.co.uk (V.B.); 3 Previous Institution: Institute of Virology, University of Bonn Medical Centre, 53127 Bonn, Germany; dr.benjamin.meyer@gmail.com (B.M.); lina.gottula@charite.de (L.T.G.); 4 Department of Theoretical and Applied Biology, Kwame Nkrumah University of Science and Technology, PMB, UPO, Kumasi 00233, Ghana; 5 Department of Medical Laboratory Technology, Kwame Nkrumah University of Science and Technology, PMB, UPO, Kumasi 00233, Ghana; 6 Institute of Virology, Charite, Universitätsmedizin Berlin, 10117 Berlin, Germany; 7 Department of Animal Science, Kwame Nkrumah University of Science and Technology, PMB, UPO, Kumasi 00233, Ghana; 8 School of Veterinary Medicine, Kwame Nkrumah University of Science and Technology, PMB, UPO, Kumasi 00233, Ghana; raphfolitse@yahoo.com; 9 Department of Agroforestry, Kwame Nkrumah University of Science and Technology, PMB, UPO, Kumasi 00233, Ghana; olivia_agbenyega@yahoo.com; 10 Department of Wildlife and Range Management, Kwame Nkrumah University of Science and Technology, PMB, UPO, Kumasi 00233, Ghana; kobbyoppong@yahoo.com

**Keywords:** Coronavirus, ELISA, livestock, intermediate host, immunofluorescence

## Abstract

Known human coronaviruses are believed to have originated in animals and made use of intermediate hosts for transmission to humans. The intermediate hosts of most of the human coronaviruses are known, but not for HCoV-NL63. This study aims to assess the possible role of some major domestic livestock species as intermediate hosts of HCoV-NL63. We developed a testing algorithm for high throughput screening of livestock sera with ELISA and confirmation with recombinant immunofluorescence assay testing for antibodies against HCoV-NL63 in livestock. Optimization of the ELISA showed a capability of the assay to significantly distinguish HCoV-NL63 from HCoV-229E (U = 27.50, *p* < 0.001) and HCoV-OC43 (U = 55.50, *p* < 0.001) in coronavirus-characterized sera. Evaluation of the assay with collected human samples showed no significant difference in mean optical density values of immunofluorescence-classified HCoV-NL63-positive and HCoV-NL63-negative samples (*F* (1, 215) = 0.437, *p* = 0.509). All the top 5% (*n* = 8) most reactive human samples tested by ELISA were HCoV-NL63 positive by immunofluorescence testing. In comparison, only a proportion (84%, *n* = 42) of the top 25% were positive by immunofluorescence testing, indicating an increased probability of the highly ELISA reactive samples testing positive by the immunofluorescence assay. None of the top 5% most ELISA reactive livestock samples were positive for HCoV-NL63-related viruses by immunofluorescence confirmation. Ghanaian domestic livestock are not likely intermediate hosts of HCoV-NL63-related coronaviruses.

## 1. Introduction

The importance of cronaviruses as emerging zoonotic viruses became evident after the international public health threat caused by severe acute respiratory syndrome coronavirus (SARS-CoV) in 2002/2003 [1]. Thereafter, there have been several studies that looked for novel coronaviruses aimed at assessing their zoonotic potential [2,3,4,5]. Coronaviruses are members of the order Nidovirales and family Coronaviridae which are made up of single-stranded positive sense RNA genomes and infect both mammalian and avian hosts. They are divided into four genera namely *Alphacoronavirus*, *Betacoronavirus*, *Gammacoronavirus*, and *Deltacoronavirus* [6,7]. In 2003, a coronavirus belonging to the *Alphacoronavirus* genus was discovered in an infant in the Netherlands and was designated human coronavirus NL63 (HCoV-NL63) [8]. This, among other coronaviruses, namely human coronavirus 229E (HCoV-229E), human coronavirus OC43 (HCoV-OC43), human coronavirus HKU1 (HCoV-HKU1), Middle East respiratory syndrome coronavirus (MERS-CoV), and SARS-CoV predominantly cause respiratory disease [1,9,10]. Human coronavirus NL63 has a worldwide distribution and is known to be associated with both upper and lower respiratory tract infections in both adults and children with seroconversion occurring at a very early age [11,12]. Most of the known human coronaviruses are believed to have originated from mammalian reservoirs such as bats and used other mammalian hosts as intermediate hosts before ending up in the human population. Some of these, like HCoV-229E and MERS-CoV, used camelid species [13,14,15,16], while SARS-CoV went through Himalayan palm civets as intermediate hosts [17,18]. Further, HCoV-OC43 is reported to have originated directly from cattle [19]. Unlike these groups of coronaviruses, HCoV-NL63 and HCoV-HKU1 have no known intermediate mammalian hosts. Human coronavirus NL63 is known to use the same receptor as SARS-CoV [20], and may therefore, like some SARS-CoV-related viruses, be capable of infecting swine [21]. This assertion is, however, yet to be explored through surveillance data. Different serological studies have mainly employed enzyme-linked immunosorbent assay (ELISA) and immunofluorescence assay (IFA) approaches for investigating HCoV-NL63 [22,23,24,25]. Most of these assays are designed for specific purposes ranging from seroprevalence studies to studies of the HCoV-NL63 genome [12,26], and would therefore vary in parameters like sensitivity and specificity. There is no single assay that is widely accepted as the standard for serological detection of HCoV-NL63, and this presents a challenge in the general validation of new assays. Coronaviruses have the potential to recombine to produce new viruses [27], and as such, knowledge of potential hosts other than humans that can be infected by two human coronaviruses is important to provide information on potential sources of novel human coronaviruses that may later spillover into human populations and cause disease. Knowledge of potential intermediate hosts of human coronaviruses will also provide information on the evolution of coronaviruses in general and interspecies transmission events that lead to emergence. The purpose of this study was therefore to assess the potential of domestic livestock species as intermediate hosts for HCoV-NL63.

## 2. Materials and Methods 

### 2.1. Study Sites

Commercial and household livestock farms across Ghana were targeted and a purposive sampling strategy was adopted. Target farms were shortlisted and visited to engage and sensitize the farm owners, family, and workers as well as the entire community. During the sensitization visits, the objectives of the study, the study design, and information on use of data was provided to potential participants. Participants were allowed to ask questions and were further encouraged to seek clarification on issues they were not convinced about.

### 2.2. Characteristics of Study Participants

The respondents in the study were sampled from both commercial and household farms. The median age of participants in the study was 34 years (range 13 to 77 years) and majority of participants were below the age of 40 years (*n* = 153, 61.7%). No ages were recorded for 5 people who either did not know or were not willing to indicate their ages. The ratio of male to female participants in the study was 194 (78.2%) males to 51 (20.6%) females, and the sexes of 3 people (1.2%) were not recorded (Table 1).

### 2.3. Collection of Serum Samples 

Serum samples were obtained from livestock farmers and their family members to be used as reference samples for assay evaluation. This was done after consent was obtained from the participants. For livestock, 10 mL of whole blood was collected and for the humans 5 mL was collected. This was done by trained veterinary technicians and clinical phlebotomists, respectively. Blood samples were then transported to the laboratory where they were centrifuged to obtain serum and immediately frozen with liquid nitrogen.

### 2.4. Algorithm for Determination of Seropositivity and Considerations for Testing

We developed a whole-virus enzyme linked immunosorbent assay (ELISA) to test for HCoV-NL63 in livestock as part of a two-stage testing algorithm also involving a recombinant immunofluorescence assay. For a sample to be considered positive for HCoV-NL63, it had to be in the top 5% most reactive samples as determined with the whole-virus ELISA and also positive in a confirmatory test with a more specific recombinant immunofluorescence assay (rIFA) [28]. This was the procedure adopted for swine, sheep, and goat sera. This ELISA relied on bovine products in the form of fetal calf serum in cell culture and milk powder for blocking and dilution of sera, and as such, cattle sera were tested directly with the recombinant immunofluorescence assay that had been optimized with less bovine products in the testing process to minimize background signals. Few donkey samples were obtained, and these were also tested directly with the recombinant immunofluorescence assay. A selection of coronavirus-characterized serum samples was used for assay optimization and the study samples for evaluation. All serum samples were heat inactivated at 56 °C for 30 min before testing.

### 2.5. Development of Whole Virus ELISA

#### 2.5.1. HCoV-NL63 Virus Culture for ELISA Antigen

High-titer virus stocks of HCoV-NL63 were produced by growing the wild-type virus (kindly provided by Lina Gottula from the lab of Prof. Christian Drosten) on Rhesus monkey kidney epithelial cells (LLC-MK2). This was done by firstly growing the cells to about 80% confluence in 162 cm^2^ cell culture flasks. A 1:17 dilution (*v/v*) of high-titer virus stock in 10-mL serum-free medium (Gibco™, Thermo Fisher, Waltham, MA, USA) was prepared and used to infect the cells for 1 h at 37 °C. Fresh Dulbecco’s Modified Eagle Medium (Gibco™, Thermo Fisher, US) supplemented with 10% Fetal calf serum (FCS) was added after infection. The flasks were incubated at 37 °C in 5% CO_2_ and harvested on day seven. 

#### 2.5.2. Virus Concentration by Ultracentrifugation 

High-titer virus stocks were produced by ultracentrifugation using a 20% sucrose cushion. Centrifugation was done on an SW 32 Ti rotor (Beckman Coulter, Brea, CA, USA) at 32,000 rpm for 4 h in vacuum and at 4 °C. The virus pellet was resuspended in 1 mL 1× phosphate buffered saline (PBS) and kept at 4 °C for 24 h to enable the pellet dissolve fully. 

#### 2.5.3. Virus Inactivation 

The ultracentrifuge-purified virus was inactivated in a 6-well tissue culture plate with 0.1% beta-Propiolactone (ACROS Organics^TM^, Thermo Fisher, US). This was done overnight at 4 °C and further incubated at 37 °C in a cell culture incubator to hydrolyze the beta-Propiolactone. The virus was then grown on LLC-MK2 cells and checked by quantitative real-time PCR for virus growth. 

#### 2.5.4. Viral Protein Quantification 

The amount of protein in the virus stock was quantified using the Bradford assay as previously described [29]. Briefly, the viral protein and a two-fold serial dilution of a protein standard (bovine serum albumin, Carl Roth, Karlsruhe, Germany) in Sodium carbonate (NaCO_3_) buffer were mixed with Bradford solution (Coomassie Plus^TM^, Thermo Fischer, US) and incubated at room temperature for 10 min. Protein quantity was subsequently measured on a spectrophotometer (Eppendorf, Hamburg, Germany) at 595 nm.

#### 2.5.5. Western Blot Analysis

For Western blot analysis, 21 μL of the ultracentrifuge purified viral protein was treated with 7 μL of NuPAGE^®^ Laemmli sample buffer (4×) (Thermo Fisher, US) and heated on a heating block at 99 °C for 5 min with rocking at 400 revolutions per minute. This was then used for sodium dodecyl sulfate polyacrylamide gel electrophoresis (SDS-PAGE) along with a recombinant HCoV-NL63 virus derived from transfected LLC-MK2 whole cell lysate. The separated proteins were then electroblotted onto a polyvinylidene difluoride (PVDF) membrane of 0.2 μm pore size (Thermo Fisher, US) and blocked with 5% milk powder dissolved in 0.1% PBS-Tween^®^ 20 (PBS-T). Following incubation with rabbit anti-Nucleocapsid and rabbit anti-Membrane primary antibodies (kindly provided by Lia van der Hoek, Department of Medical Microbiology, University of Amsterdam), the membrane was washed with PBS-T and incubated with horseradish peroxidase (HRP)-conjugated goat anti-rabbit immunoglobulin G (Cell Signaling Technology, Danvers, MA, USA) for 1 h at room temperature. The membrane was subsequently analyzed with a chemiluminescent substrate, SuperSignal West Femto Maximum Sensitivity Substrate kit (Thermo Fisher, US) according to the manufacturer’s instructions.

#### 2.5.6. Optimization of ELISA Protocol

Conditions for sample testing were determined by testing several combinations of conditions using the protocol of an in-house recombinant MERS-CoV ELISA as reference (Appendix A) and adopting the most optimal conditions. Dilution series of antigen ranging from 12 µg/mL to 0.1875 µg/mL and conjugate ranging from 1:500 to 1:4000 were tested to determine the optimal coating concentration and optimal conjugate dilution, respectively. The determined optimal conjugate dilution for human testing was used as a starting concentration for determination of optimal conjugate dilution for livestock testing. Coronavirus-characterized sera previously tested for HCoV-NL63 by a recombinant immunofluorescence assay were used as positive and negative test sera and were tested in replicates at a starting dilution of 1:100 then compared to 1:200 for assay optimization. These sera were also in combination either positive or negative for HCoV-229E and HCoV-OC43 (Table 2) and were used to assess potential cross reactivity with other coronaviruses. These serum samples were obtained from the lab of Prof. Christian Drosten. Different substrate exposure times were also tested to determine the most optimal duration. 

#### 2.5.7. Final ELISA Testing Procedure

In brief, viral protein was coated into 96-well Nunc MicroWell^TM^ plates (Thermo Fisher, US) by diluting the stock to 0.75 µg/mL in NaCO_3_ buffer (0.1 M, PH 9.6) and coating with 50 µL per well with overnight incubation at 4 °C. The plates were washed 5 times with 0.1% PBS-T then blocked with 5% milk powder (Carl Roth, Germany) in PBS-T for one hour at room temperature and the wash repeated. Serum to be tested were diluted 1:200 in 1% milk powder in PBS-T. After one-hour incubation at room temperature, plates were washed 5 times with PBS-T and goat anti-human antibody labelled with Horseradish peroxidase enzyme (Dianova GmbH, Hamburg, Germany) at a dilution of 1:2000 was added. For livestock testing, HRP-coupled donkey anti-sheep, goat anti-swine, and donkey anti-goat antibodies (Dianova GmbH, Hamburg, Germany) were used for sheep, swine, and goat testing, respectively, also at a 1:2000 dilution. The conjugate was incubated at room temperature for 30 min after which plates were washed 5 times with PBS-T and an enzyme substrate, 3,3′,5,5′-Tetramethylbenzidine (TMB) (Mikrogen Diagnostik, Neuried, Germany) was then added. This was kept in the dark for 15 min and stopped with 2 Molar sulphuric acid (H_2_SO_4_) and the absorbances read at 450 nm and 630 nm on a Biotek synergy 2 (BioTek Instruments Inc., Winooski, VT, USA) multi-detection plate reader.

### 2.6. Recombinant Spike Immunofluorescence Testing

Screening by recombinant immunofluorescence assay was conducted as previously described [30]. Briefly, Vero B4 cells were transfected with pCG1 eukaryotic expression vectors bearing the complete HCoV-NL63 spike sequence. Transfected cells were incubated overnight after which cells were harvested and spotted onto multi-test slides (12 spots, 5 mm diameter, Dunn Labortechnik GmbH, Asbach, Germany), fixed with ice-cold acetone/methanol and stored dry at 4 °C until use. Serum samples were tested at a 1:40 dilution for 1 h at 37 °C, which was optimal for reducing nonspecific reactions and maintaining sensitivity. Secondary detection was performed with Alexa Fluor 488-conjugated goat anti-human antibody (Dianova GmbH, Hamburg, Germany) for human testing. For livestock testing, Alexa Fluor 488-conjugted goat anti-bovine, goat anti-horse, goat anti-swine, donkey anti-sheep, and donkey anti-goat antibodies (Dianova GmbH, Hamburg, Germany) were used to test cattle, donkey, swine, sheep, and goat sera, respectively. These secondary antibodies have previously been confirmed to work on the tested species [28,31]. Each sample was spotted onto transfected and non-transfected cells to help distinguish autofluorescence from fluorescence due to immune reactions.

### 2.7. Ethical Issues

Ethical approval for the study was obtained from the committee on human research, publications and ethics of the school of medical sciences, Kwame Nkrumah University of Science and Technology (Protocol number CHRPE49/09). Permission for livestock sampling was also obtained from the wildlife division of the Ghana Forestry Commission (Approval Number: AO4957).

### 2.8. Data Analysis

Descriptive graphs were generated using Microsoft Excel and IBM Statistical Package for Social Sciences (SPSS) version 20. After subtraction of plate background, differences in rIFA-categorized mean optical densities were assessed by one-way analysis of variance (ANOVA) and percentiles were used to determine the most ELISA reactive samples for both human and livestock samples using SPSS. A Mann–Whitney U test was used to compare the mean ranks of optical density values of HCoV-NL63-rIFA-characterized sera for assay optimization. The number of samples with optical density values above the 75th, 80th, 85th, 90th, and 95th percentile optical density values constituted the top 25, 20, 15, 10, and 5 percent most reactive samples respectively. Map data was plotted with Tableau public 10.5 and a livestock distribution beeswarm plot was generated using R statistical package version 3.4.3.

## 3. Results

### 3.1. Distribution of Samples Collected

**Figure 1:**
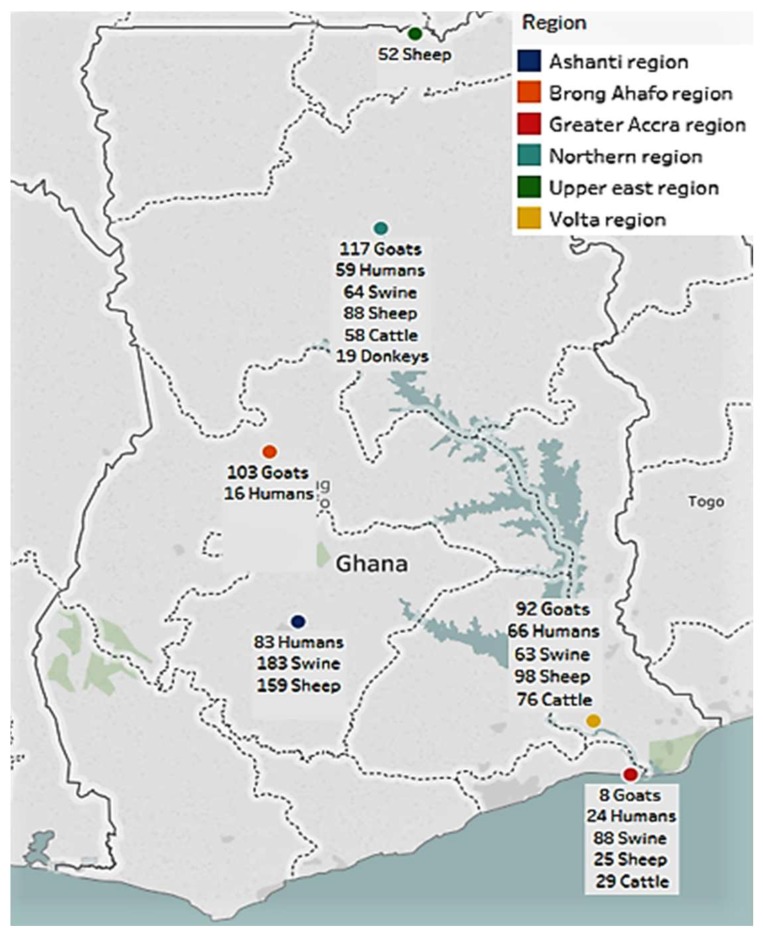
Distribution of human and livestock samples collected in the study.

A total of 248 people was sampled from five different regions of Ghana. The majority of people sampled were in the Ashanti region (*n* = 83, 33.5%) and the fewest in the Brong Ahafo region (*n* = 16, 6.5%). A total of 1321 serum samples from 397 pigs, 422 sheep, 320 goats, 163 cattle, and 19 donkeys was collected in the study. The majority of swine samples (*n* = 182, 45.8%) and sheep samples (*n* = 159, 37.7%) was collected from the Ashanti region. The Northern region was the source of majority of the goat samples (*n* = 117, 36.6%) as well as all 19 donkey samples and majority of the cattle samples were obtained from the Volta region (*n* = 76, 46.6%) (Figure 1).

### 3.2. Analysis of Virus Protein

#### Western Blot Analysis

**Figure 2:**
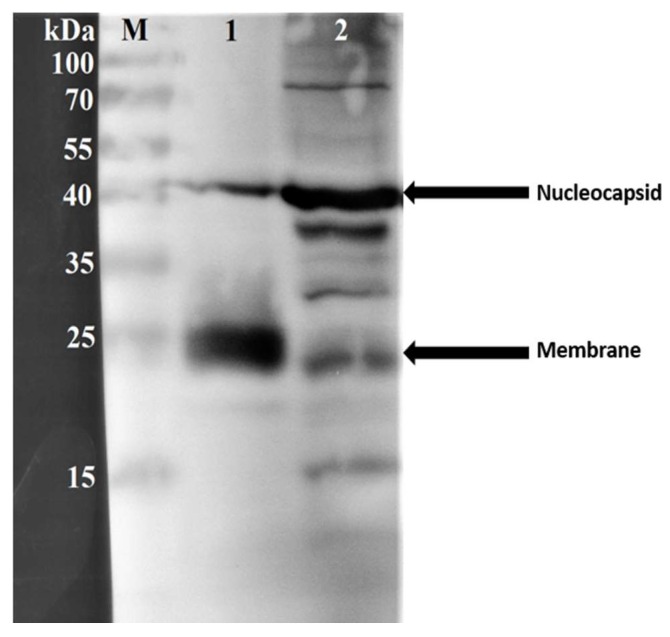
Identification of HCoV-NL63 Nucleocapsid (N) and Membrane (M) proteins. Western blot analysis of the purified HCoV-NL63 antigen (lane 1) compared to HCoV-NL63 protein recovered from transfected LLC-MK2 whole cell lysate (lane 2). Lane M shows the molecular weight standard in Kilodaltons.

Western blot analysis was performed to confirm the identity and immunogenicity of two major structural proteins of HCoV-NL63 in the concentrated virus antigen. The viral antigen obtained after ultracentrifugation and inactivation showed the nucleocapsid protein (N) around the 40 kilodalton mark and membrane (M) protein around the 25 kilodalton mark (Figure 2). The band sizes for the nucleocapsid protein for the transfected LLC-MK2 whole cell lysate-derived control was at a similar position (~40 kilodaltons) but was more prominent than that of the test virus protein. The membrane protein of the control was, however, less prominent than that of the test virus protein (Figure 2) indicating a likelihood of higher composition of whole virion particles in the concentrated virus protein antigen. The approximate molecular masses of the two structural proteins and their detection by the respective primary antibodies confirmed their identity and immunogenicity. 

### 3.3. Determination of ELISA Testing Conditions

**Figure 3:**
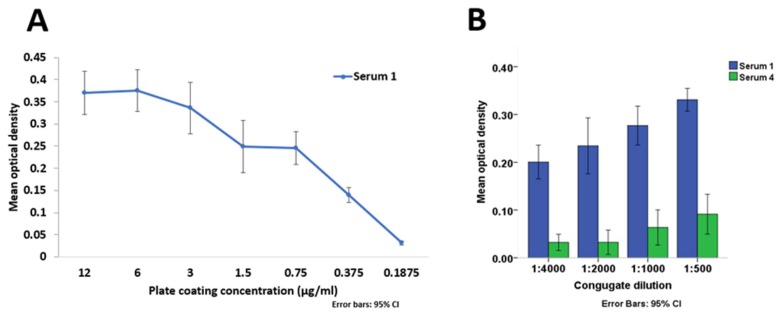
Mean optical densities of HCoV-NL63-rIFA-positive serum with coating concentrations ranging from 12 µg/mL to 0.1875 µg/mL (Panel (**A**)). Mean optical densities for the combined 5- and 15-min substrate incubation times for HCoV-NL63 rIFA-positive (Serum 1) and negative (Serum 4) sera with different conjugate dilutions (Panel (**B**)). Error bars depict 95% confidence intervals.

A variable range of plate coating concentrations were assessed to determine saturation point in order to inform selection of an appropriate coating concentration. There was a two-fold reduction in average optical density (OD) measured using the HCoV-NL63-rIFA-positive test serum on coating concentration range 0.75 µg/mL to 0.1875 µg/mL (Figure 3A) depicting the range of consistent detectable variation. There was no consistent variation in average OD of the HCoV-NL63-rIFA-positive serum sample tested with coating concentrations exceeding 0.75 µg/mL (Figure 3A), and as such, this value was chosen as the coating concentration for the assay. 

An increase in the mean OD of a combined 5- and 15-min incubation time for serum 1 from 1:4000 to 1:500 dilution was observed. There was no increase in mean OD from 1:4000 to 1:2000, but from 1:2000 to 1:500 dilution for serum 4 (Figure 3B). The 1:2000 dilution was selected for testing human samples due to the low reactivity of the negative serum 4 despite the increase in reactivity of the positive serum 1. 

**Figure 4:**
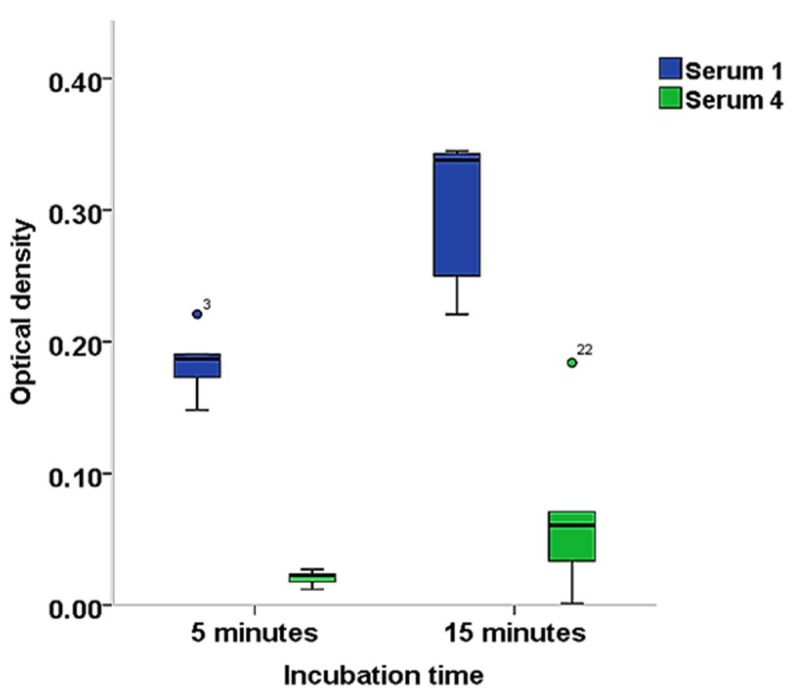
Distribution of optical densities comparing 5- and 15-min substrate incubation times for HCoV-NL63 rIFA-positive (Serum 1) and negative (Serum 4) sera. Colored dots indicate outliers.

The 15-min substrate incubation time produced higher reactivity for serum 1 with a median (interquartile range) OD of 0.338 (0.236 to 0.344) compared to the 5-min incubation time with 0.187 (0.163 to 0.191). The reactivity was similarly higher for the 15-min incubation time with a median (interquartile range) OD of 0.06 (0.025 to 0.099) than the 5-min incubation time with 0.022 (0.016 to 0.024) for serum 4 (Figure 4). The 15-min incubation time was selected for higher sensitivity. 

**Figure 5:**
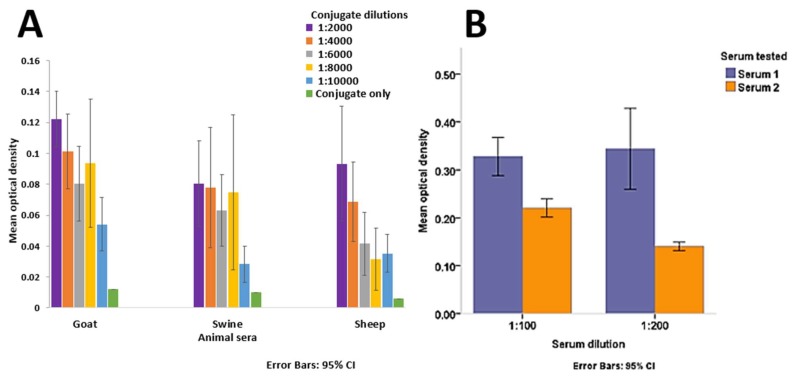
Mean optical densities of livestock sera at different conjugate dilutions (Panel (**A**)). Mean optical density comparison for HCoV-NL63-positive (Serum 1) and negative (Serum 2) sera at two dilutions (Panel (**B**)). Error bars depict 95% confidence intervals.

The 1:2000 conjugate dilution was also found to be optimal for livestock sera testing in terms of reactivity to noise comparison with a higher difference in mean OD between livestock sera and wells tested with conjugate only compared to other dilutions (Figure 5A). 

A 1:200 serum dilution was chosen for serum testing as this gave a better discrimination between the positive and negative serum samples compared to a 1:100 dilution (Figure 5B). The further dilution of the serum provided better detection of the target protein in the positive serum and lower reactivity in the negative serum as observed in Figure 5B.

### 3.4. Potential Cross Reactivity with Other Coronaviruses 

**Figure 6:**
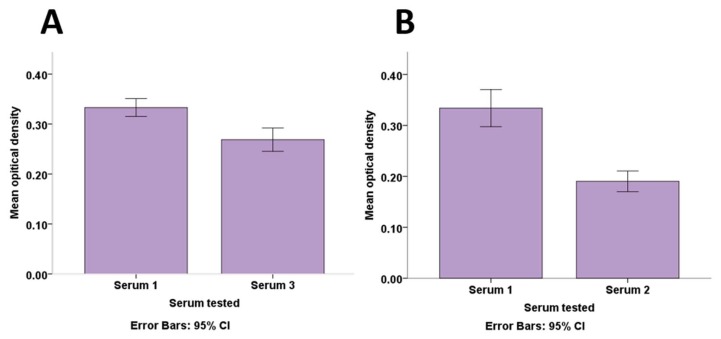
Comparison of mean optical density values of HCoV-NL63-rIFA-positive (Serum 1) and HCoV-NL63-rIFA-negative (Serum 2 and 3) sera also positive for HCoV-229E (Serum 3) (Panel (**A**)) and HCoV-OC43 (Serum 2) (Panel (**B**)) showing a difference between the positive and negative test sera. Error bars indicate a 95% confidence interval.

The ability of the assay to specifically detect and significantly differentiate HCoV-NL63 in samples co-infected with other coronaviruses was assessed. Likelihood of false detections in samples that were HCoV-NL63 negative but positive for other coronaviruses was also assessed. Different degrees of cross-reactivity with HCoV-229E and HCoV-OC43 were observed with a higher level seen with HCoV-229E than with HCoV-OC43 (Figure 6). There was a statistically significant difference (U = 27.50, *p* < 0.001) as determined by the Mann–Whitney U test in mean ranks of optical density between serum 1 (mean OD = 0.33) which was positive by rIFA for both HCoV-NL63 and HCoV-229E and serum 3 (mean OD = 0.27) which was negative for HCoV-NL63 but positive for HCoV-229E (Figure 6A). The difference in optical density values of serum 1 which was positive for HCoV-NL63 and HCoV-OC43 and serum 2 (mean OD = 0.19) which was negative for HCoV-NL63 but positive for HCoV-OC43 (Figure 6B) was also statistically significant (U = 55.50, *p* < 0.001). This indicates the assay’s capability of distinguishing HCoV-NL63 from these coronaviruses.

### 3.5. Evaluation of ELISA with HCoV-NL63-rIFA Test of Human Samples

**Figure 7:**
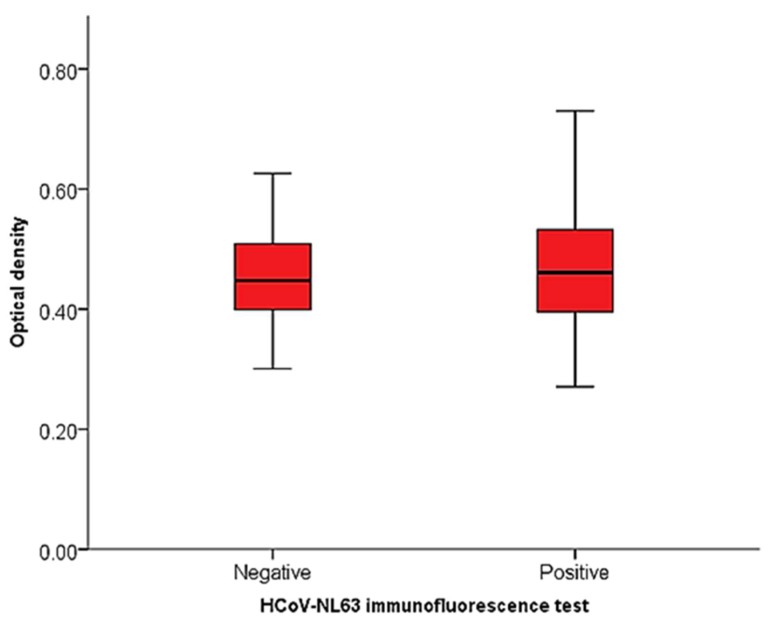
Distribution of optical density values of HCoV-NL63-rIFA-tested human samples.

After testing the 248 human samples using the immunofluorescence assay, 217 samples were determined to be unequivocal positives and negatives by two independent assessors. These were samples that did not produce significant background noise and autofluorescence to hinder result determination and were used in analysis. Out of this number, 183 (84.3%) were positive and 34 (15.7%) were negative. Optical density values for the human sample testing ranged from 0.27 to 0.73 for the HCoV-NL63-rIFA-positive samples and 0.30 to 0.63 for HCoV-NL63-rIFA-negative samples (Figure 7). A comparison of the mean OD values of rIFA positive (0.47 ± 0.10) and negative (0.46 ± 0.09) groups showed no statistically significant difference between the groups as determined by one-way ANOVA (*F* (1, 215) = 0.437, *p* = 0.509). Assay validation parameters like sensitivity and specificity could not be reliably estimated as a result of the lack of an available gold standard assay for HCoV-NL63 serology. A gradual increase in the ratio of HCoV-NL63-rIFA positives to negatives with an increase in cut point OD by percentiles was observed. All the top 5% most ELISA reactive samples were HCoV-NL63-rIFA-positive as compared to the top 25% most reactive samples of which only 84% were HCoV-NL63-rIFA-positive (Table 3) indicating a higher probability of the most ELISA reactive samples testing positive by immunofluorescence testing. 

### 3.6. HCoV-NL63 in Livestock Samples

**Figure 8:**
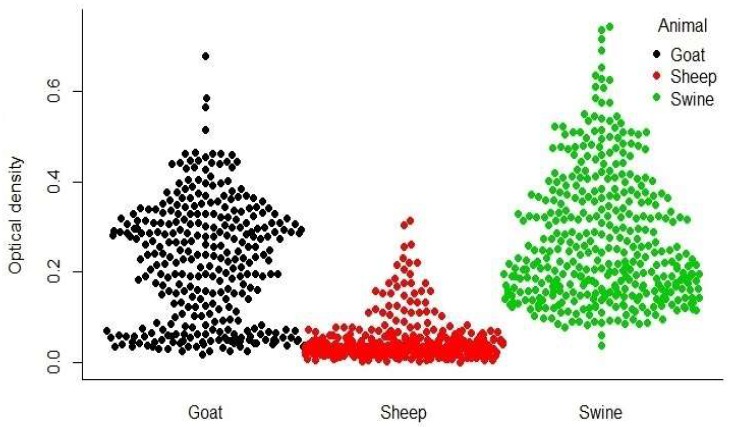
Distribution of optical density values of livestock sera.

In order to determine the livestock ELISA reactivity patterns and the most reactive sheep, goat, and swine samples, these livestock species were subjected to screening with the developed ELISA. The optical density values for the livestock testing ranged from 0.0 to 0.32 for sheep, 0.02 to 0.68 for goats, and 0.04 to 0.74 for swine (Figure 8). None of the most reactive swine, sheep, and goat sera as determined by the 95th percentile OD cut point tested positive by rIFA. Donkeys (*n* = 19) and cattle (*n* = 163) that were tested for HCoV-NL63 by rIFA were all negative as well (Table 4). Given the relatively large number of samples tested across species and the lack of positivity, these livestock species sampled in Ghana are not likely to be intermediate hosts for HCoV-NL63.

## 4. Discussion

For the purpose of sero-surveillance in an effort to detect antibodies from Ghanaian cattle, sheep, goats, swine, and donkeys against HCoV-NL63-related viruses, an indirect, whole-virus ELISA was developed in this study as part of a two-stage testing algorithm. This was done in order to assess the possibility of any of the previously mentioned species being an intermediate host for this virus. The recombinant immunofluorescence assay described in this study is a robust, sensitive, and specific assay [32]. The assay is, however, time consuming and requires an experienced person to interpret results, and as such, is not suitable for use on a large scale. Purification of HCoV-NL63 antigen through the sucrose medium is a method that has previously been used for HCoV-NL63 [24] and other coronaviruses [33,34] and has been found to be an effective method of antigen purification and concentration as was seen in this study as well. Although the sucrose cushion is not as effective as the density gradient for the separation of complete from incompletely assembled virion particles [35], the sucrose cushion used in this study appeared to be sufficiently effective for this purpose. 

The signal comparison for the positive and negative test samples was adequate for discrimination despite the negative sample being positive for HCoV-229E; which belongs to the same serologic group as HCoV-NL63, and for HCoV-OC43 which belongs to the other group of the two serologic groups of human coronaviruses. Lack of cross-reactivity between HCoV-NL63 and the more closely related HCoV-229E as well as with HCoV-OC43 has been reported by other studies that employed recombinant ELISAs targeting the nucleocapsid protein [12,36]. Despite the fact discrimination was possible in the present study using the whole virus antigen, some degree of cross-reactivity was also observed. 

Apart from cross-reactivity with the other coronaviruses, antibodies may cross-react with other unrelated proteins in the serum. The sera used in optimizing the ELISA and the tested samples had different demographic characteristics and as such the level of reactivity may differ in the tested samples compared to the samples used for optimization. This is however useful given the assay was to be eventually used for testing sera from different species to the one used for optimization and evaluation. The highly reactive samples, however, are more likely to be positive for the target of interest as was observed in this study and other studies as well [32,37].

The number of known positive and negative samples used in evaluation affects the likely diagnostic sensitivity and specificity of a candidate assay [38]. In the present study, fewer negative than positive samples were obtained for the evaluation of the assay as a result of the sample being taken from a cross-section of the population where the eventual test subjects were also obtained albeit of a different species. The fewer negatives obtained in the cross-section and used in the evaluation is likely to result in a less accurate assessment of diagnostic specificity. The purpose of the present assay did not, however, require a highly accurate measure of specificity, but interest was geared towards sensitivity. These parameters were, however, not estimated due to the lack of a gold standard assay.

Being the main immunogenic structural proteins of coronaviruses, the nucleocapsid, spike, and membrane proteins are important in assay development [39,40,41]. The nucleocapsid protein is produced abundantly during infection and is employed in assay development because it is a potent immunogen [40,42]. One study on SARS-CoV showed the nucleocapsid induced the production of antibody levels comparable to the whole virus and slightly higher than the spike protein [43]. The reactivity pattern observed in the present study with the full virus antigen will comprise a collective effect of specific and non-specific interactions of serum antibodies with the three main immunogenic structural proteins and other protein moieties. Although the nucleocapsid protein is the most abundantly produced during infection with HCoV-NL63 [44,45], the membrane protein is more abundant in the complete virion particle than nucleocapsid protein [46,47]. This was seen in the Western blot analysis after ultracentrifugation with the sucrose cushion which evidently concentrated more complete virion particles. The immune responses observed are likely to be mainly due to the membrane protein because of its abundance in the whole virus antigen.

For simple in-house preparations, the indirect ELISA is a good choice and also provides high sensitivity and flexibility [48,49]. The limitations with this process include possibility of high background signal due to the binding of all proteins to the wells of ELISA plates and non-specific binding of the secondary antibody [50]. The competitive ELISA technique has an added advantage of no requirement for sample clean-up and a high sensitivity to differences in composition of complex mixtures of different antigens even in the presence of relatively small quantities of the specific detection antibody [51,52]. Whole virus antigen preparations like the one used in this study have generally been found to be more sensitive than recombinant antigen targets [53,54] but tends to be less specific as a result of higher likelihood of non-specific binding of co-purified cellular proteins and non-target viral proteins [55,56].

Although several bat species have been found to harbor several alpha and betacoronaviruses believed to be the ancestors of endemic human coronaviruses including HCoV-NL63 [13,23], bats may not have been a direct source of virus transmission to humans given that, CoVs such as SARS-CoV and MERS-CoV both make use of terrestrial mammals which are more likely to have contact with humans instead of bats as transmission hosts. Again, HCoV-229E is more closely related to their relatives in camels as compared to those in bats, indicating a probability of camels being intermediate hosts between bats and humans [14,57]. Human coronavirus NL63 uses the angiotensin-converting enzyme (ACE) 2 receptor for infection of target cells similar to SARS-CoV [20] and has been found to be able to replicate in swine cells in vitro [58]. No antibodies to HCoV-NL63 were found in any of the pigs tested in the present study as evidence of the fact that the ability to replicate in swine cells does not imply capability to infect an actual animal since several other barriers need to be surmounted for this to happen. Based on the results of the present study it may be concluded that cattle, sheep, goats, donkeys, and swine may not be intermediate hosts for HCoV-NL63. However, there have not been any reports of HCoV-NL63-related viruses circulating in Ghanaian bats, and as such, a spillover opportunity may not be present, and hence no likely infection. Surveillance of local livestock populations can also be performed for antibodies in areas where such HCoV-NL63-related viruses have been detected like in Kenya to confirm this [57]. 

Coronaviruses have been shown to have the potential to mutate and genetically recombine when two viruses infect the same cell [59], as seen for instance with recombination events between canine coronavirus and transmissible gastroenteritis virus and canine coronavirus and feline coronaviruses that have brought about new coronaviruses [60,61]. These new viruses may have a different host range particularly if changes occur in the spike region or different pathology in the same host and as such knowing the possible intermediate hosts of coronaviruses that infect humans is important as these provide information on the evolution of the virus as well as possible mixing vessels for these viruses.

## Figures and Tables

**Table 1:** Characteristics of human samples collected in the study.

Characteristic	Number	Percent
Age categories (years)		
10–44	171	69
45–80	72	29
Missing	5	2.0
Sex		
Male	194	78.2
Female	51	20.6
Missing	3	1.2

**Table 2:** Profile of Coronavirus-characterized sera.

Serum ID	Origin	rIFA Testing		
		HCoV-NL63	HCoV-229E	HCoV-OC43
Serum 1	Germany	+	+	+
Serum 2	China	−	−	+
Serum 3	Germany	−	+	+
Serum 4	Germany	−	+	+

HCoV: Human coronavirus; ID: Identification; rIFA: Recombinant immunofluorescence assay; +: Positive; −: Negative.

**Table 3:** Proportions of rIFA positives among the most ELISA reactive human samples by percentile cut point.

Cut Point Percentile	Cut Point Optical Density	Number of Samples with OD above Cut Point	rIFA Result	
			Positive *n* (%)	Negative *n* (%)
75th	0.54	50	42 (84)	8 (16)
80th	0.55	38	33 (86.8)	5 (13.3)
85th	0.58	26	23 (88.5)	3 (11.5)
90th	0.61	18	16 (88.9)	2 (11.1)
95th	0.64	8	8 (100)	0 (0)

OD: Optical density; rIFA: Recombinant immunofluorescence assay; *n*: Number; %: Percentage.

**Table 4:** Confirmation of most reactive livestock samples with HCoV-NL63-rIFA.

Livestock	Number	95th Percentile Cut Point	Number of Samples with OD above Cut Point	rIFA Result
Goat	320	0.44	16	All negative
Swine	397	0.53	19	All negative
Sheep	422	0.15	21	All negative
Donkey	19	-	-	All negative
Cattle	169	-	-	All negative

OD: Optical density; rIFA: Recombinant immunofluorescence assay.

## Appendix A

**MERS ELISA-Testing protocol**

- Coating: Coat Antigen on Nunc MicroWell™ MaxiSorp™ Plates (Sigma–Aldrich, M9410 Sigma) diluted in NaCO_3_ buffer (0.1 M, PH 9.6) 50 µL/well O/N at 4 °C
- Antigen: 0.1% β-propiolactone-inactivated MERS CoV (3 µg/mL in NaCO_3_ buffer)
- Washing: 5× with 100 µL/well PBS/0.1% Tween [PBS/T]
- Blocking: 100 µL/well PBS/T-5% milk powder → 1 h
- Washing: 5× with 100 µl/well PBS/0.1% Tween [PBS/T]
- Sera: Test dilutions at 100 µL/well: 1:50, 1:200, 1:800 (Screening human sera: 1:400 should be fine) for 1 h.
  ∘ Dilution in PBS/T-1% milk powder
  ∘ Negative controls: ONLY PBS/T-1% milk
  ∘ Positive control: 1:800
- Washing: 5× with 100 µL/well PBS/0.1% Tween [PBS/T]
- Conjugate: anti-human IgG HRP labeled 1:4000 in PBS/T-1% milk for a maximum of 1 h (100 µL/well)
- Washing: 5× with 100 µL/well PBS/0.1% Tween [PBS/T]
- Substrate: 100 µL/well (TMB, Microgen). Keep out of light. The reaction is stopped with 2 M H_2_SO_4_ (100 µL/well).
  ∘ The time is dependent on the sera. Positive human sera should be detectable within 3–5 min. In case there is no signal after 30 min, you can be sure the test is negative.
- Read: ELISA-Reader protocol “Screening ELISA”:
  ∘ 450 nm yellow color
  ∘ 630 nm background plates
- Calculation: The 630 nm values (plate background) were subtracted from the 450 nm (sample) values
  ∘ Negative control: secondary antibody only, or better if possible, a negative serum (Should also be negative for other Coronaviruses like HCoV-OC43)
  ∘ Subtract negative control mean value from the other values
